# Supplementary material for: Selfish mutations promote age-associated erosion of mtDNA integrity in mammals
Source: Nat Commun. 2025 Jul 1;16:5435. doi: 10.1038/s41467-025-60477-y (PMC12216451; doi:10.1038/s41467-025-60477-y)
Supplement: Supplementary file 1 — Supplementary Information [file 41467_2025_60477_MOESM1_ESM.pdf]

Supplementary Information

Selfish mutations promote age-associated erosion of mtDNA integrity in mammals

Ekaterina Korotkevich[1,*], Daniel N. Conrad[2], Zev J. Gartner[2], Patrick H. O'Farrell[1,*]

[1]Department of Biochemistry and Biophysics, University of California, San Francisco, San Francisco, California, USA

[2]Department of Pharmaceutical Chemistry, University of California, San Francisco, San Francisco, California, USA

*Correspondence: ekaterina.korotkevich@ucsf.edu, ofarrell@cgl.ucsf.edu

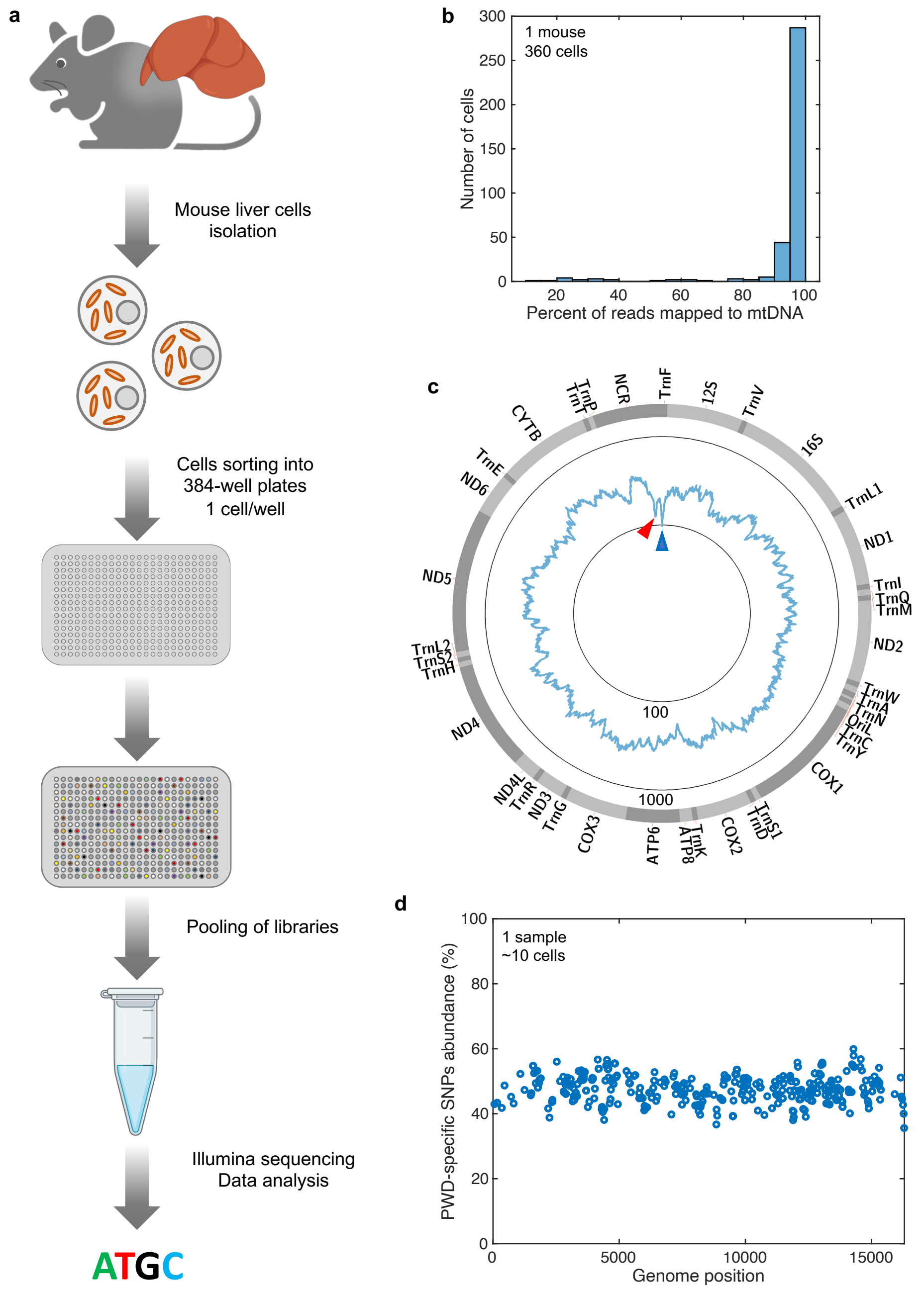

Supplementary Figure 1. Plate-based single-cell mtATAC method workflow and performance. a Schematic of the plate-based single-cell mtDNA sequencing workflow. Parts of the schematic were created in BioRender. Korotkevich, E. (2025) https://BioRender.com/s42y778. **b** Histogram showing the percentage of raw reads mapping to mtDNA as measured for 360 cells isolated from 24-month-old C57BL6/J WT mouse liver. The majority of reads map to mtDNA. **c** Average mtDNA coverage as measured for 360 cells isolated from 24-month-old C57BL6/J WT mouse liver (log10 scale). Although this method allows profiling the entire mtDNA, coverage is not uniform. Coverage in NCR is notably lower than in other regions of mtDNA. The coverage drop at the genome breakpoint (blue arrowhead) is caused by alignment to a linearized reference, whereas the coverage drop between positions 16100 and 16170 (red arrowhead) is likely due to inaccessibility resulting from binding of the region by replication/transcription machinery. **d** Control showing the assessed abundance of PWD-specific SNPs in a sample where equal number of C57BL6/J and mtPWD hepatocytes were mixed (n ≤ 5 cells per sample type, the uncertain cell number is due to inefficient FACS sorting). In this single sample all polymorphic sites should be present at equal levels. The variation in abundances shows the level of precision of mutation abundance detection.

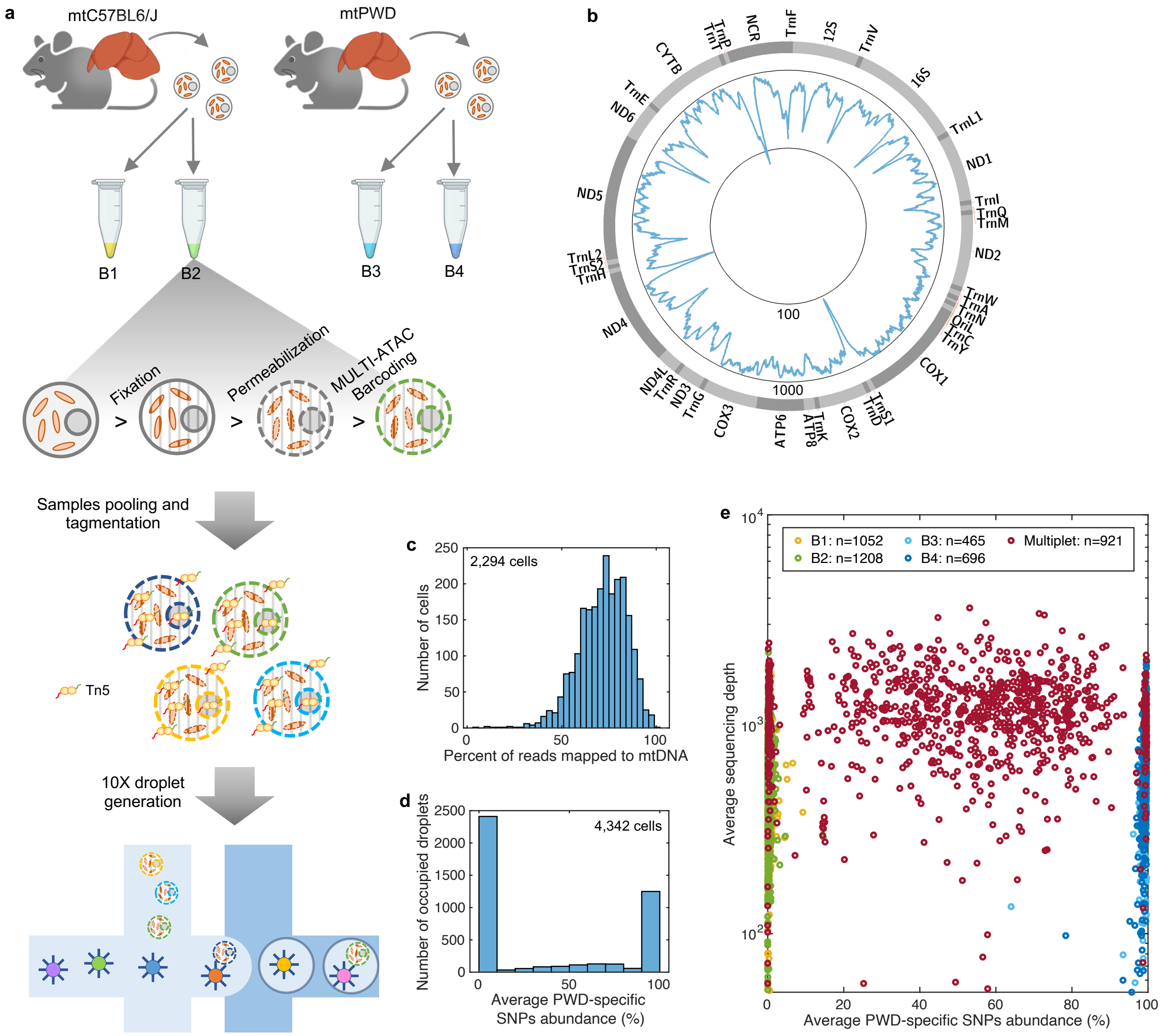

Supplementary Figure 2. Test of the performance of the multiplex-10X-method. a Schematic of the proof-of-principle experiment. Hepatocytes from two mouse lines with distinct mtDNA sequences (mtC57BL6/J and mtPWD) were fixed and permeabilized. Each biological sample was split into two halves and each of the four resulting samples was labeled with a unique MULTI-ATAC barcode (B1-B4). MULTI-ATAC labeled samples were pooled together, tagmented and loaded onto 10X chip to generate 10X-mtATAC and MULTI-ATAC barcode libraries. In this experiment, there are two independent markers of sample origin, the MULTI-ATAC barcodes and the polymorphisms distinguishing mtC57BL6/J from mtPWD. A high degree of correspondence between the two markers would indicate the accuracy of the multiplexing method. Parts of the schematic were created in BioRender. Korotkevich, E. (2025) https://BioRender.com/p92g415. **b** Average mtDNA coverage in single mtC57BL6/J cells (log10 scale). Coverage is not uniform across the mtDNA but is sufficient for detection of mutations at most positions. **c** A histogram showing enrichment of mtDNA reads over total reads in single mtC57BL6/J cells. The high degree of enrichment of mtDNA reads makes the pipeline efficient and minimizes possible contamination by nuclear mitochondrial DNA segments (NUMTs). The percent of reads mapping to mtDNA is higher than previously reported (e.g., Lareau et al., 2021) likely due to much higher mtDNA copy number in hepatocytes compared to human hematopoietic cell lines. **d** A histogram of the number of occupied droplets carrying different percentages of PWD-specific SNPs. The peaks on the left and on the right represent droplets carrying mtC57BL6/J and mtPWD cells, respectively. A shallow and wide distribution in the middle represents droplets with more than one cell (multiplets) carrying both mtC57BL6/J and mtPDW cells. **e** Classification of droplets according to sample-specific MULTI-ATAC barcodes matches cells' mtDNA genotype. Droplets marked only by B1 or B2 align at the left consistent with mtC57BL6/J cells, while droplets marked with B3 or B4 align at the right consistent with mtPWD cells. There are three kinds of multiplet droplets: homotypic ones carrying both B1 and B2, which have only mtC57BL6/J cells; homotypic ones carrying B3 and B4 which have only mtPWD cells; and heterotypic ones carrying either B1 or B2 together with either B3 or B4, which have both types of cells. Most of the data points are on the left and right extremes (see **d**) and homotypic multiplets are masking underlying single cell data. Mean PWD-specific SNPs abundance for B1 and B2 singlets (mtC57BL6/J cells) is 0.35%, mean PWD-specific SNPs abundance for B3 and B4 singlets (mtPWD cells) is 99.12%. Inset shows number of droplets.

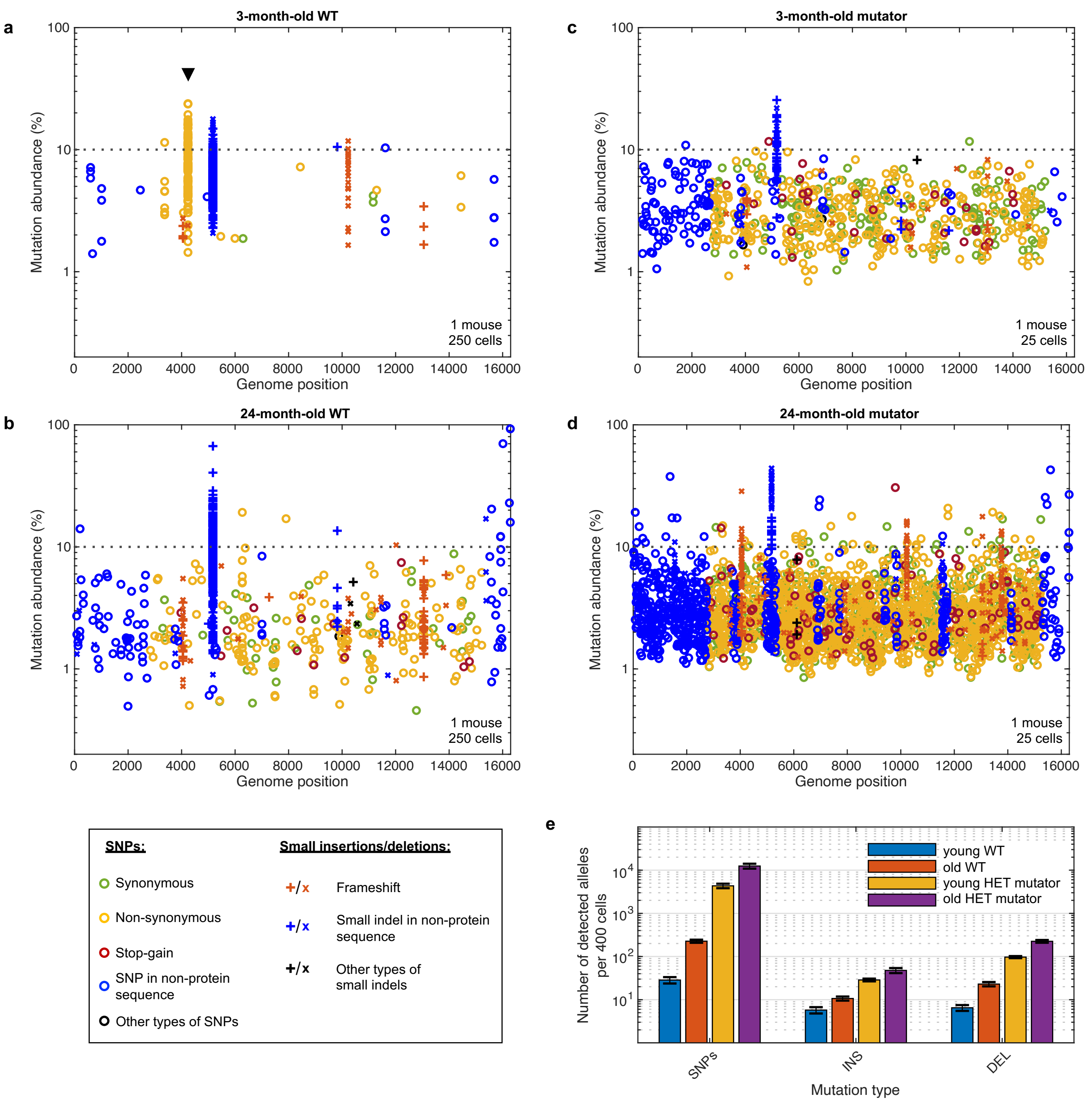

Supplementary Figure 3. Spectrum of mtDNA mutations in WT and heterozygous mutator mouse livers. a Plot of all mutations detected in 250 liver cells isolated from of a 3-month-old C57BL6/J WT mouse. The arrowhead marks a clonally expanded mutant allele. Y-axis is logarithmic, which allows better visualization of low abundance mutations. Note that the lack of data towards the bottom of the distribution (below 1% abundance) reflects the detection sensitivity limitation rather than the actual frequency of low-abundance mutations, which simulations predict would be very high. **b** Plot of all mutations detected in 250 liver cells isolated from a 24-month-old C57BL6/J WT mouse. This is the same dataset that is presented in Fig. 1c. **c** Plot of all mutations detected in 25 liver cells isolated from a 3-month-old heterozygous mutator mouse. **d** Plot of all mutations detected in 25 liver cells isolated from a 24-month-old heterozygous mutator mouse. This is a subset of the dataset presented in Fig. 1f. **a-b**, The dotted line marks 10% abundance. **e** Number of detected mutant alleles per 400 liver cells reported separately for SNPs, small insertions (INS) and small deletions (DEL). Samples from four young (3-month-old) and three old (24-month-old) C57BL6/J WT mice, as well as three young and three old heterozygous mutator mice, were analyzed. For this analysis data were subsampled to 100,000 mtDNA-mapped reads per cell. This approach allows for fair comparison between samples but reduces the detection power of low abundance mutations. The number of detectable mutant alleles increased with age.

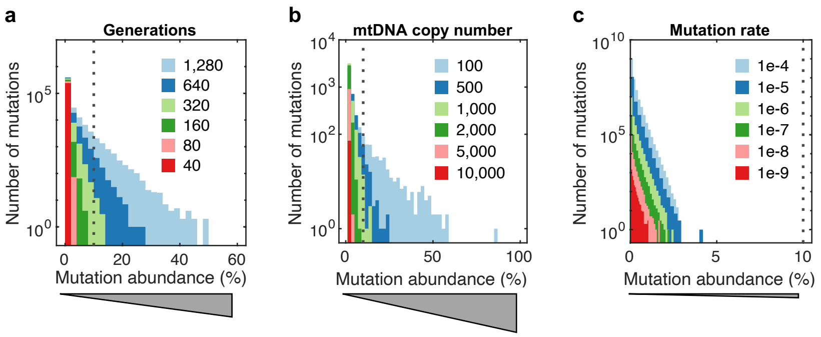

Supplementary Figure 4. Neutral mtDNA mutations are unlikely to reach impactful levels. Simulations illustrating the impact of key variables on the abundance distribution of mutations: time (number of Generations) (**a**), mtDNA copy number (**b**) and mutation rate (**c**). Grey wedges highlight difference in X-axis scale for **a-c** panels. The dotted line marks 10% abundance. Model parameters, unless otherwise specified in the figure panels, are as follows: 16,299 bp genome, 10,000 genomes/cell, mutation rate $2x10^{-8}$/bp/replication cycle, 40 Generations, 10,000 simulated cells. In the lifetime of a mouse (~40 Generations), the accumulation of a neutral mutation to 10% abundance in a cell with a high mtDNA copy number (~10,000 copies) is exceedingly unlikely.

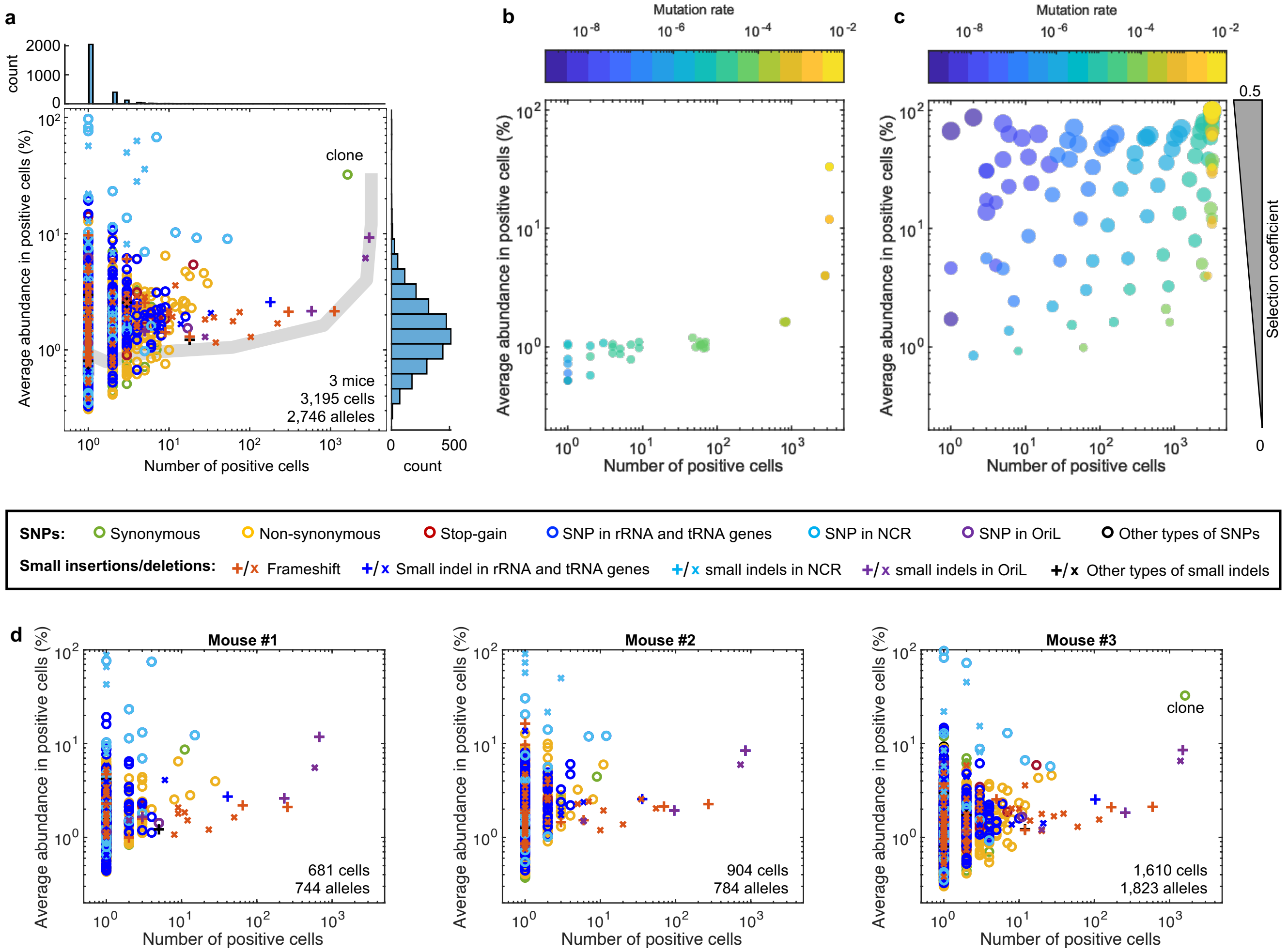

Supplementary Figure 5. Allele behavior on AAA vs C# plots. a An AAA vs C# scatter plot showing the **a**verage cellular **a**bundance of each mutant **a**llele (AAA) in the positive cells (Y-axis) versus the number of cells (C#) in which the allele was detected (X-axis). Accompanying histograms show the abundance distribution of alleles (right) and the frequency with which alleles are detected among analyzed cells (top). Data shown for 24-month-old C57BL6/J mouse livers (the same dataset as in Fig. 1d,e and Supplementary Fig.3e, but obtained from an independent experiment from the one shown in Fig. 3a). The grey line shows expected location of neutral alleles emerging at varying rates. **b**,**c** Simulations showing positions of neutral alleles emerging at differing mutation rates (**b**) or alleles differing in both mutation rate and selection coefficient (**c**) on AAA vs C# plots. Mutation rate (per bp per replication cycle) and selection coefficient are indicated by color and size scales, respectively. Model parameters: 10,000 genomes/cell, 40 Generations, 3,195 cells. **d** AAA vs C# plots for each individual mouse presented in panel **a**.

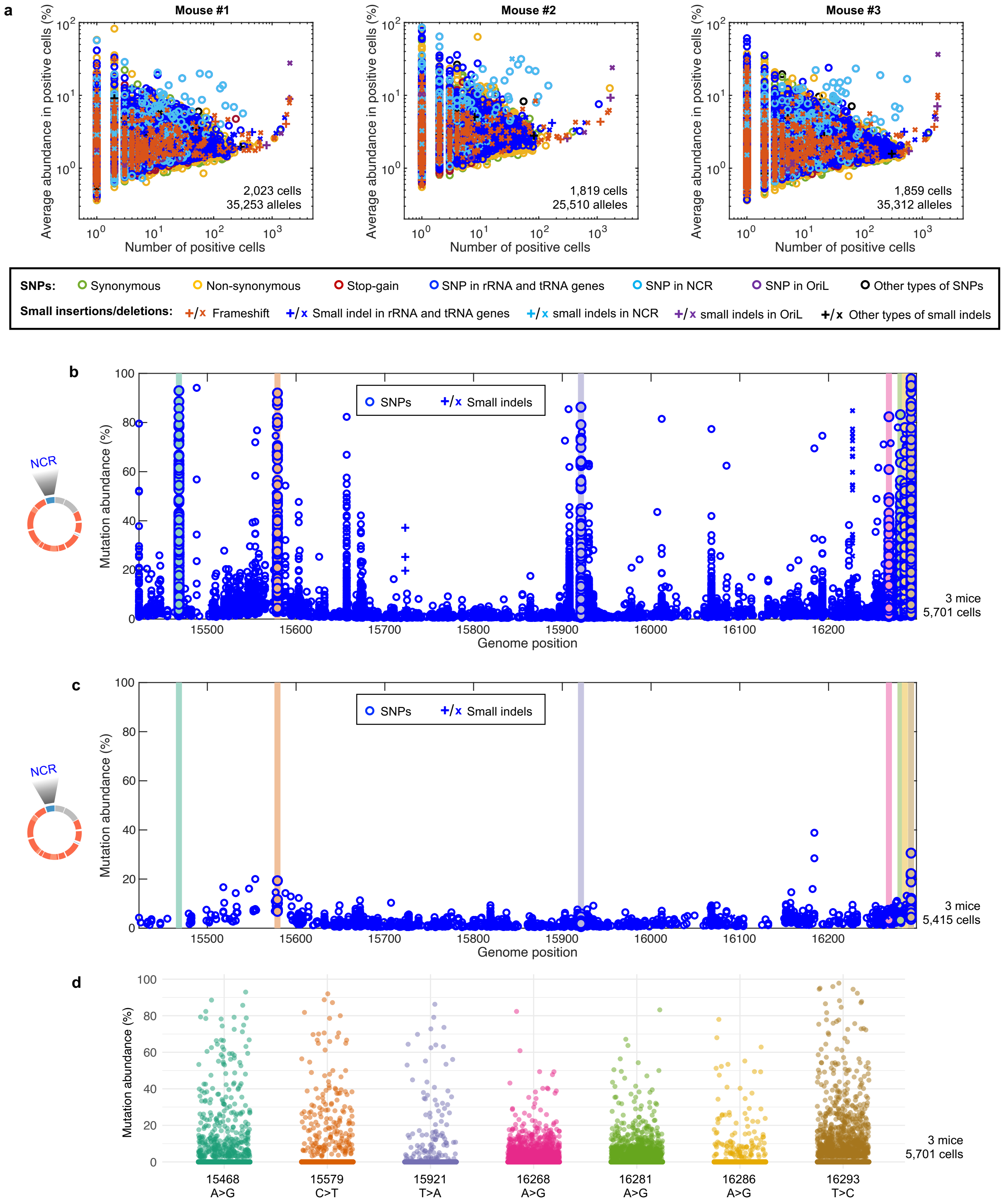

Supplementary Figure 6. Specific mutations in the NCR of heterozygous mutator mice confer a competitive advantage. **a** AAA vs C# plots for hepatocytes from three 24-month-old heterozygous mutator mice. **b** Spectrum of mutations in the NCR of liver cells from 24-month-old heterozygous mutator mice. Mutations present at over 20% abundance in at least three cells in all three mice are highlighted with color bars and marker filling. **c** Spectrum of mutations in the NCR of liver cells from 3-month-old heterozygous mutator mice. **d** Abundance distribution of specified NCR mutations among liver cells of 24-month-old heterozygous mutator mice presented in panel **a** and **b**. Note a peculiar distribution of abundance of 16268A>G and 16281A>G mutations: these mutations are detected in many cells, however in the majority of cells these variants stay below 10% abundance, reaching high levels in only a few cells. One possible explanation for this atypical abundance distribution is that these variants confer a selective advantage only in a small subset of liver cells.

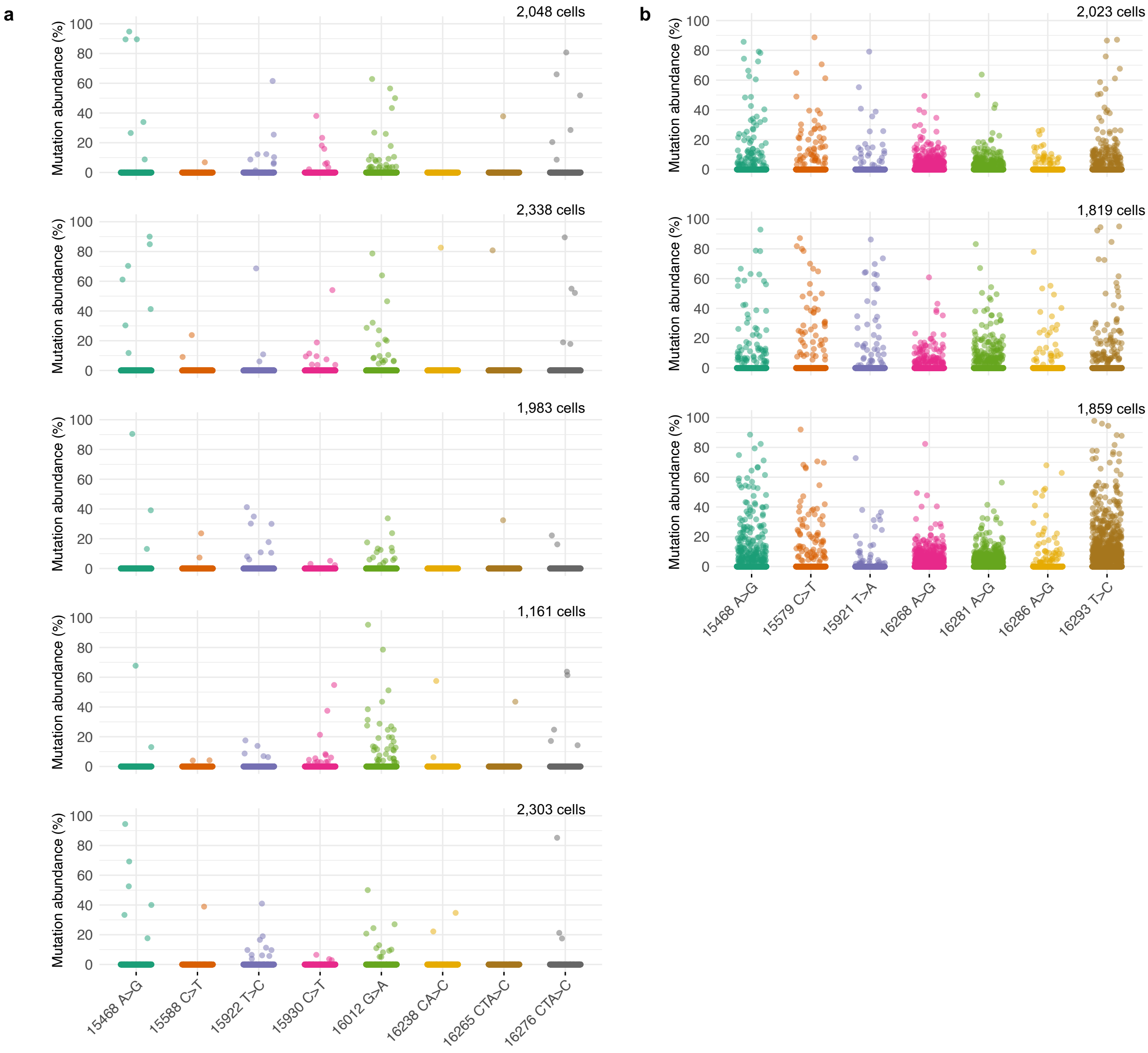

Supplementary Figure 7. Abundance of driver mutations in liver cells. Cellular abundance of driver alleles in 24-month-old WT (**a**) and heterozygous mutator (**b**) mouse livers. Each plot shows data from an individual mouse.

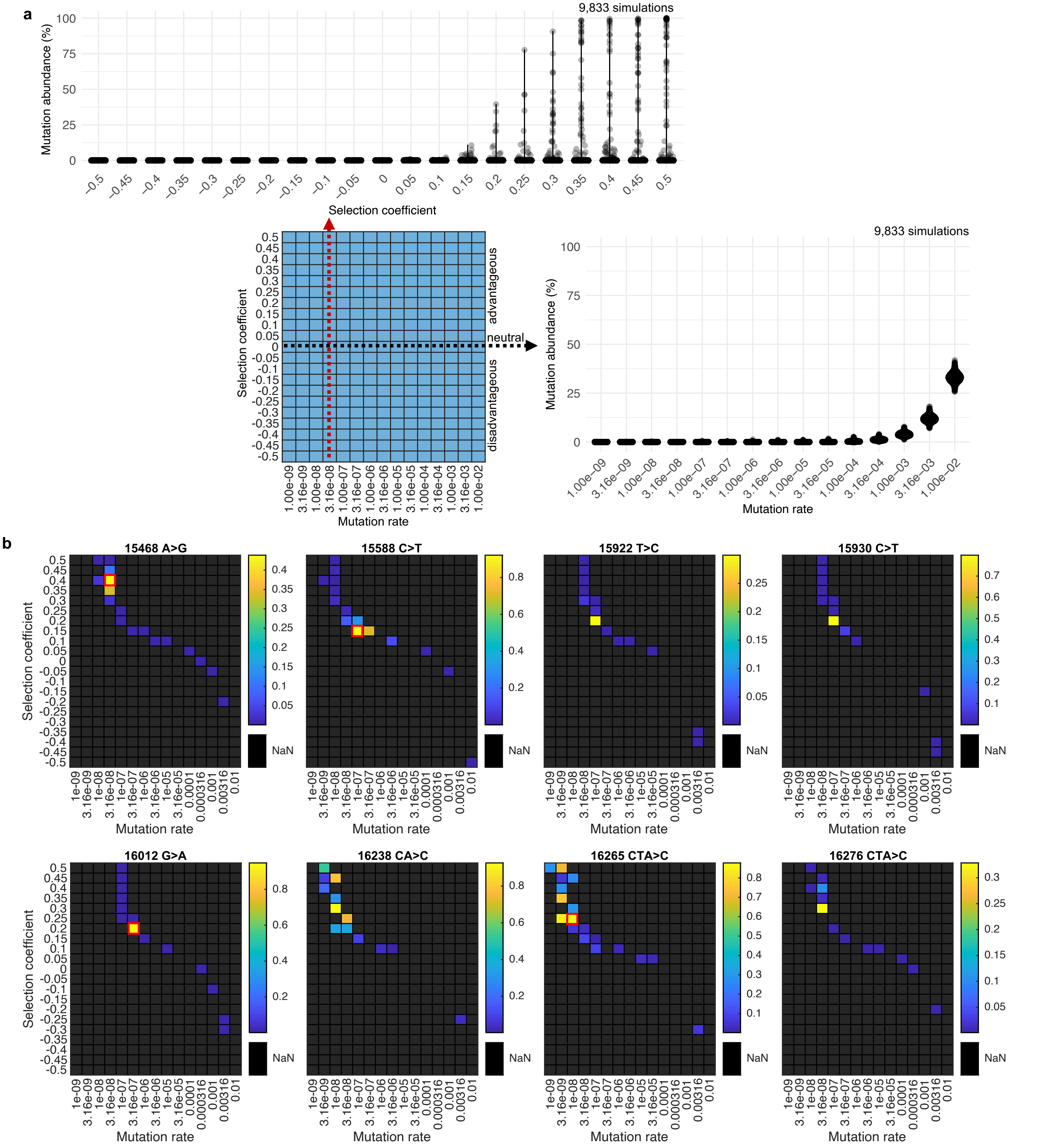

Supplementary Figure 8. Modeling recurrent NCR mutations in WT mouse liver. a The impact of a range of mutation rates and selection coefficients on mutant allele abundance distribution among cells was explored in simulations. Top panel shows how selection coefficient impacts abundance distribution of a mutant allele emerging at rate of 3.16×10^{-8} per bp per replication cycle. Right panel shows how mutation rate impacts the abundance distribution of a neutral mutant allele. The results are shown for simulations that ran for 40 Generations that we estimate to be equivalent to mtDNA turnover over 2 years of mouse life. Number of mtDNAs per simulated cell was set to 10,000. **b** Heatmaps showing similarity between abundance distribution of specified driver alleles and simulation outcomes. Colors represent "similarity value" (mean difference p-value based on 1,000 permutations) with yellow shades signifying the strongest similarity (see Methods for details). Black squares represent parameter sets that produced 2-fold more or 2-fold less cells with detected mutant allele than real data. N=9,833 simulated cells. The squares framed in red mark the parameter set used in comparing the distributions created by simulations to experimentally determined abundance distributions in Fig. 3e.

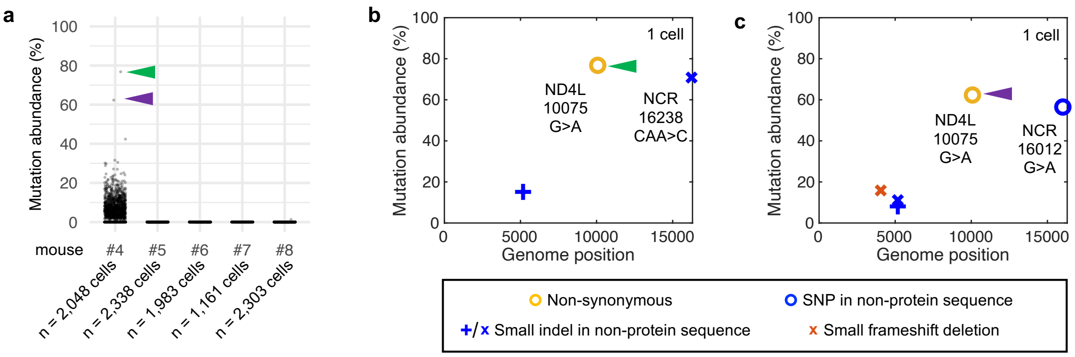

Supplementary Figure 9. Clonal mutations are frequent passengers. a 10075G>A was detected in many cells of one of five mice analyzed in an experiment. This sporadic occurrence indicates that 10075G>A is either a germline variant or a mutation that emerged early in development. Among the many cells positive for 10075G>A, two cells had this mutation at exceptionally high abundance (green and purple arrowheads). This dataset is the same as in Fig. 3a. **b**,**c** Mutational spectra of the cells with exceptionally high abundance of 10075G>A. In both cells a NCR mutation is present at similar levels to 10075G>A. Note that 16238CAA>C is a distinct allele from 16238CA>C reported in Fig. 3. This allele was observed only once in our dataset and therefore did not meet the conservative thresholds for driver alleles. However, its location in the NCR and its high abundance in the affected cell, which cannot be explained otherwise, suggest that 16238CAA>C is a driver with a very low mutation rate. Notably, among three cases of driver-passenger linkage identified in this mouse (only mutations with >50% abundance were considered), two included the clonally expanded allele.

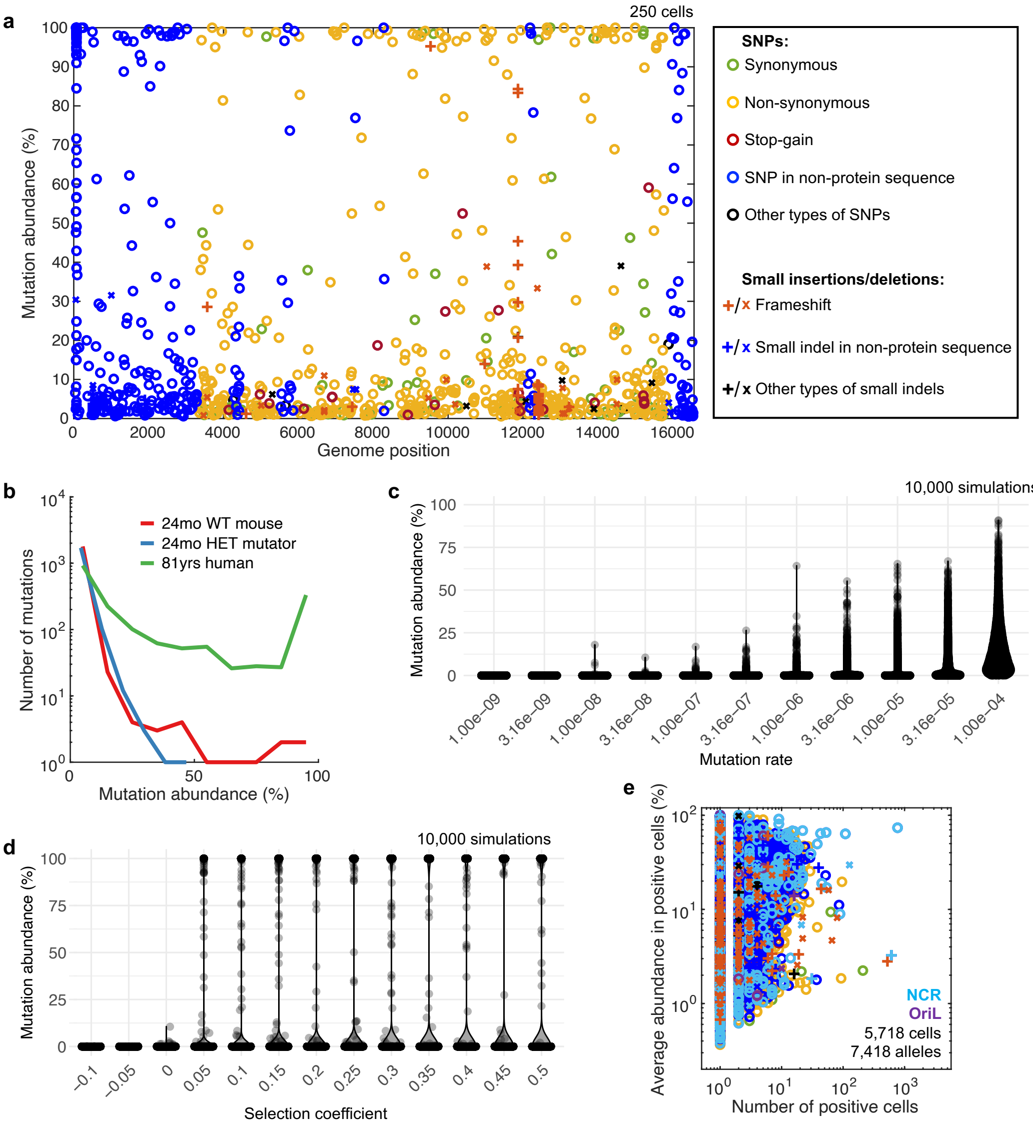

Supplementary Figure 10. Spectrum of mtDNA mutations and modeling of mtDNA mutation accumulation in aged human liver cells. a A spectrum of mtDNA mutations identified in hepatocytes from an 81-year-old human. **b** Abundance distribution of mutations in hepatocytes from 24-month-old WT mice (three mice from one experiment), 24-month-old heterozygous mutator mice (three mice from one experiment) and an 81-year-old human. Data from each sample were subsampled to an equal number of reads per cell (100,000). For mouse data, clonal mutations and indels in OriL were excluded since they are very frequent and mask the signal from other mutations. Samples were normalized to have an equal number of mutations: 1,829 mutations were randomly selected for each sample. These data indicate an increased mutation rate has little influence on the proportion of mutations reaching high abundance, whereas age has a large impact. **c** Impact of mutation rate (per bp per replication cycle) on the abundance distribution of a neutral mutant allele among cells. The results are shown for simulations that ran for 1,600 Generations that we estimate to be equivalent to mtDNA turnover over 81 years of human life. Number of mtDNA copies per cell was set to 5,000. **d** The impact of the selection coefficient on the abundance distribution of a mutant allele among cells. Notably, even a modest selection coefficient is sufficient to drive mutations to 100% abundance. Model parameters: 5,000 genomes/cell, mutation rate 3.16×10^{-8} per bp per replication cycle, 1,600 Generations. **e** AAA vs C# plot for all sequenced hepatocytes from an 81-year-old human.

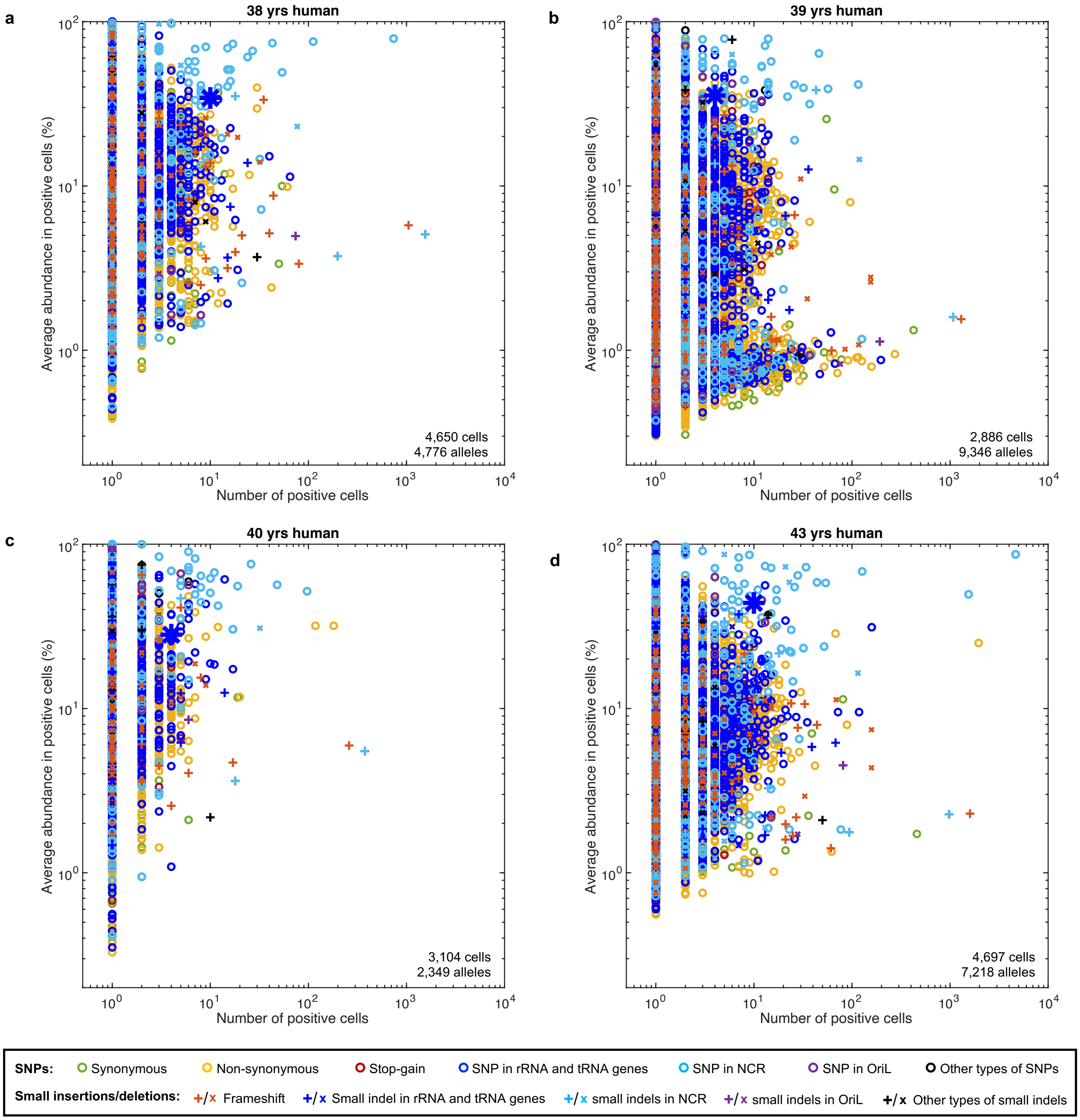

Supplementary Figure 11. mtDNA mutation spectra in hepatocytes from middle-age humans. a-d AAA vs #C plots for liver cells from middle-aged humans. The 3243A>G allele colocalizes with NCR driver alleles and is marked by a large blue asterisk. Differences in the number of alleles with low AAA across samples are due to variations in coverage.

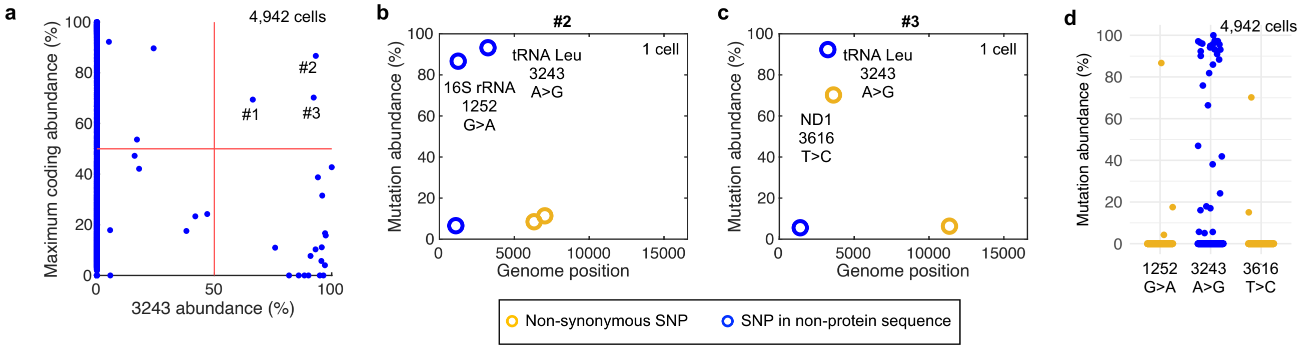

Supplementary Figure 12. Passenger mutations on mtDNAs with 3243A>G mutation. **a** Maximum abundance of 3243A>G versus maximum abundance of coding alleles (excluding 3243A>G) in a cell plotted for all sequenced liver cells isolated from a 41-year-old human. In cells located in the top-right quadrant of the graph, 3243A>G is linked to another mutation in a coding sequence. Data for cell #1 are presented in Fig. 5h. Data for cells #2 and #3 are presented in panels **b** and **c**. **b**,**c** Mutational spectra of cells where 3243A>G is linked to a passenger mutation. For cell #3, it is likely that multiple genomes carried 3243A>G before 3616T>C emerge on one of these mutant genomes. **d** Abundance distribution of 1252G>A, 3243A>G and 3616T>C among all sequenced liver cells from the 41-year-old human.
