# Supplementary material for: Selfish mutations promote age-associated erosion of mtDNA integrity in mammals
Source: Nat Commun. 2025 Jul 1;16:5435. doi: 10.1038/s41467-025-60477-y (PMC12216451; doi:10.1038/s41467-025-60477-y)
Supplement: Supplementary file 2 — Description of Additional Supplementary Files [file 41467_2025_60477_MOESM2_ESM.pdf]

Description of Additional Supplementary Files

Supplementary Data 1. mtDNA variants detected in hepatocytes of 24-month-old WT mice. A list of mtDNA variants, their mean abundance in positive cells, and the number of positive cells among analyzed hepatocytes from 24-month-old WT mice. Aggregated data across multiple mice as well as data for individual mice are provided in separate tabs.

Supplementary Data 2. Human drivers. Limited clinical information on de-identified human hepatocyte donors and a list of mtDNA alleles classified as drivers in these samples. For each individual, the number of positive hepatocytes with driver variant abundance below and above 50% is reported, along with comparison to previously published data.

Supplementary Data 3. Oligo sequences. Sequences of oligonucleotides used in this study. Oligonucleotides for ddPCR, plate-based mtATAC and MULTI-ATAC applications are reported in different tab.
