# Supplementary material for: Selfish mutations promote age-associated erosion of mtDNA integrity in mammals
Source: Nat Commun. 2025 Jul 1;16:5435. doi: 10.1038/s41467-025-60477-y (PMC12216451; doi:10.1038/s41467-025-60477-y)
Supplement: Supplementary file 6 — Reporting Summary [file 41467_2025_60477_MOESM6_ESM.pdf]

nature portfolio

Corresponding author(s): Ekaterina Korotkevich
Patrick O'Farrell

Last updated by author(s): Apr 23, 2025

Reporting Summary

Nature Portfolio wishes to improve the reproducibility of the work that we publish. This form provides structure for consistency and transparency in reporting. For further information on Nature Portfolio policies, see our Editorial Policies and the Editorial Policy Checklist.

Statistics

For all statistical analyses, confirm that the following items are present in the figure legend, table legend, main text, or Methods section.

n/a	Confirmed	
☐	☒	The exact sample size (*n*) for each experimental group/condition, given as a discrete number and unit of measurement
☐	☒	A statement on whether measurements were taken from distinct samples or whether the same sample was measured repeatedly
☐	☒	The statistical test(s) used AND whether they are one- or two-sided<br>*Only common tests should be described solely by name; describe more complex techniques in the Methods section.*
☒	☐	A description of all covariates tested
☐	☒	A description of any assumptions or corrections, such as tests of normality and adjustment for multiple comparisons
☐	☒	A full description of the statistical parameters including central tendency (e.g. means) or other basic estimates (e.g. regression coefficient) AND variation (e.g. standard deviation) or associated estimates of uncertainty (e.g. confidence intervals)
☐	☒	For null hypothesis testing, the test statistic (e.g. *F*, *t*, *r*) with confidence intervals, effect sizes, degrees of freedom and *P* value noted<br>*Give P values as exact values whenever suitable.*
☒	☐	For Bayesian analysis, information on the choice of priors and Markov chain Monte Carlo settings
☒	☐	For hierarchical and complex designs, identification of the appropriate level for tests and full reporting of outcomes
☒	☐	Estimates of effect sizes (e.g. Cohen's *d*, Pearson's *r*), indicating how they were calculated

Our web collection on statistics for biologists contains articles on many of the points above.

Software and code

Policy information about availability of computer code

Data collection	only instrument's associated software was used
Data analysis	QuantaSoft Analysis Pro v.1.0.596 software (Bio-Rad), bwa v0.7.17, Picard tool v2.27.4, bamtools v2.5.2, samtools v1.16.1, Freebayes v1.3.6, bcftools v1.16, vcflib v1.0.3, IGV74,75 v 2.4.16, SnpEff v5.0, SIFT 4G, Cell Ranger ATAC v 2.1.0, deMULTIplex2 v1.0.1, SLiM v3.6, Mutation-Simulator v 3.0.1

For manuscripts utilizing custom algorithms or software that are central to the research but not yet described in published literature, software must be made available to editors and reviewers. We strongly encourage code deposition in a community repository (e.g. GitHub). See the Nature Portfolio guidelines for submitting code & software for further information.

Data

Policy information about availability of data

All manuscripts must include a data availability statement. This statement should provide the following information, where applicable:

- Accession codes, unique identifiers, or web links for publicly available datasets
- A description of any restrictions on data availability
- For clinical datasets or third party data, please ensure that the statement adheres to our policy

The sequencing data have been deposited in the NCBI Sequence Read Archive under PRJNA1146058.

Research involving human participants, their data, or biological material

Policy information about studies with human participants or human data. See also policy information about sex, gender (identity/presentation), and sexual orientation and race, ethnicity and racism.

Reporting on sex and gender	Due to limited availability both male and female human hepatocytes were used. No sex-based analyses were performed.
Reporting on race, ethnicity, or other socially relevant groupings	4 of 6 human liver donors were Caucasian, 1 donor was white without further specification if Hispanic or not, and 1 was of unknown race/ethnicity. Race, ethnicity or other socially relevant groupings were not considered in the study design.
Population characteristics	Human liver donors were 38, 39, 40, 41, 43 and 81 years old. All except 41-year-old donor were male.
Recruitment	Human hepatocytes were purchased from Xenotech, Lonza and UCSF Liver Center.
Ethics oversight	All human samples were de-identified by providers, ethical approval and oversight are not required.

Note that full information on the approval of the study protocol must also be provided in the manuscript.

Field-specific reporting

Please select the one below that is the best fit for your research. If you are not sure, read the appropriate sections before making your selection.

- [x] Life sciences
- [ ] Behavioural & social sciences
- [ ] Ecological, evolutionary & environmental sciences

For a reference copy of the document with all sections, see nature.com/documents/nr-reporting-summary-flat.pdf

Life sciences study design

All studies must disclose on these points even when the disclosure is negative.

Sample size	No statistical methods were used to predetermine sample size. There are two aspects of sample sizes: number of individuals and number of cells. To identify events that are unique to individuals we used a minimum of 3 individuals per condition. The number of cells depended on efficiency of our methods.
Data exclusions	For plate-based experiments cells with fewer than 10,000 reads mapping to mtDNA were excluded. For 10X-based experiments cells with mean mtDNA coverage below 50 were excluded. In both cases the exclusion was done to remove data from low quality cells or wells/droplets containing ambient DNA. For mtDNA copy number quantification using ddPCR samples containing fewer than 100 copies were considered negative and excluded.
Replication	For each age group tissues or cells from at least 3 individuals (independent biological replicates) were analyzed. Exceptions were mtDNA copy number measurements where two individuals per age group were used and 81-year-old human sample presented in Supplementary Fig. 10a,b,e. Each experiment was repeated at least once with the same or orthogonal method.
Randomization	Randomization is not relevant to this study as no interventions were performed and all samples were treated identically.
Blinding	Investigators were not blinded during experiments and outcome assessment. In all experiments all biological samples were treated identically, therefore blinding was not necessary.

Reporting for specific materials, systems and methods

We require information from authors about some types of materials, experimental systems and methods used in many studies. Here, indicate whether each material, system or method listed is relevant to your study. If you are not sure if a list item applies to your research, read the appropriate section before selecting a response.

Materials & experimental systems

n/a	Involved in the study	
x		Antibodies
x		Eukaryotic cell lines
x		Palaeontology and archaeology
	x	Animals and other organisms
x		Clinical data
x		Dual use research of concern
x		Plants

Methods

n/a	Involved in the study	
x		ChIP-seq
x		Flow cytometry
x		MRI-based neuroimaging

Animals and other research organisms

Policy information about studies involving animals; ARRIVE guidelines recommended for reporting animal research, and Sex and Gender in Research

Laboratory animals	The following strains of mice were used: C57BL6/J, mtPWD (C57BL/6J-mtPWD/Ph/ForeJ), heterozygous mutator (B6.129S7(Cg)-Polgtm1Prol/J). 3- to 24-month-old mice were used.
Wild animals	The study did not involve wild animals.
Reporting on sex	For WT C57BL6/J mice only males were used.<br>For heterozygous mutator mice both males and females were used due to limited number of available animals. Sex-based analyses were not performed.
Field-collected samples	The study did not involve samples collected from the field.
Ethics oversight	Experiments in this study were approved by the University of California, San Francisco Institutional Animal Care and Use Committee.

Note that full information on the approval of the study protocol must also be provided in the manuscript.

Plants

Seed stocks	*Report on the source of all seed stocks or other plant material used. If applicable, state the seed stock centre and catalogue number. If plant specimens were collected from the field, describe the collection location, date and sampling procedures.*
Novel plant genotypes	*Describe the methods by which all novel plant genotypes were produced. This includes those generated by transgenic approaches, gene editing, chemical/radiation-based mutagenesis and hybridization. For transgenic lines, describe the transformation method, the number of independent lines analyzed and the generation upon which experiments were performed. For gene-edited lines, describe the editor used, the endogenous sequence targeted for editing, the targeting guide RNA sequence (if applicable) and how the editor was applied.*
Authentication	*Describe any authentication procedures for each seed stock used or novel genotype generated. Describe any experiments used to assess the effect of a mutation and, where applicable, how potential secondary effects (e.g. second site T-DNA insertions, mosiacism, off-target gene editing) were examined.*
